# An Analysis of the New York Anti-Narcotic Law

**Published:** 1914-11

**Authors:** 


### ABSTRACTS

**An Analysis of the New York Anti-Narcotic Law.** Judge Edward Swann, of the Court of General Sessions, New York. From The Medical Times, August, 1914. As the laws enacted by the legislature during the past winter have not yet been printed in book form and the average citizen therefore has no facilities for familiarizing himself with them, it seems fitting that some workable analysis in narrative form should be made for temporary convenience of the provisions of the Anti-Narcotic Drug Law which went into effect July 1st. This matter is of special interest to physicians, surgeons, dentists, veterinarians and pharmacists.

To the drug addict and the drug peddler the only advice that can be given is to let the prohibited drugs alone. Against the illicit distributors of habit-forming narcotic drugs the law is especially directed and it is full of teeth for them.

The act in question is an amendment to the ''Public Health Law'' in relation to the sale of habit-forming drugs. It does not affect the existing Anti-Cocaine Law (section 1746 of the Penal Law) which makes it a misdemeanor to possess and a felony to sell cocaine except as therein provided. The new law is additional to and supplemental of the existing Anti-Cocaine Law and is directed against the illegal possession or use of chloral, opium, or any of its salts, alkaloids or derivatives, or any compound or preparation of any of them (which includes heroin, morphine and codeine).

Violation of the law is a misdemeanor and is punishable by one year in the penitentiary, or by a fine of $500, or by both. There seems to prevail an idea that all persons arrested for violation of this new Anti-Narcotic Drug Act shall be confined separate and apart from other prisoners while awaiting trial, but there is no such provision or suggestion made in the statute and such is not the case. All prisoners in the City Prison awaiting trial are ''presumed to be innocent until the contrary is shown,'' and they are only detained for the purpose of making sure of their presence at the trial.

Nor does the statute provide that even after conviction they shall be confined separate and apart from other prisoners except a certain class, of which I predict that in the whole State there will not be twelve such convictions in a year, viz., the habitual addict to the drug who has been so adjudged by a magistrate and committed to a hospital for treatment, and who after his commitment to the hospital is charged by the hospital authorities with violating its rules and ''repeatedly conducts himself in a disorderly manner,'' and is thereafter tried and convicted of such disorderly conduct and sentenced to the penitentiary or workhouse. In which case, such institution shall ''keep such person separate and apart from the other inmates,'' the object being not to subject the unfortunate and confirmed user of the drug to contact with criminals.

The act does not apply to the sale of proprietary medicines sold in good faith, provided they do not contain more than a certain stated limited quantity of the prohibited drugs. The intent and object of the law is to prevent the improper use of habit-forming drugs, and in order to effectually do so it has been necessary to regulate their legitimate sale and use in order that the drugs may be checked up and accounted for from the hands of the manufacturer to the consumer, and this is effected by means of a series of official order blanks furnished by the State to the local Board of Health, and by the local Board of Health to pharmacists, druggists, physicians, veterinarians, or dentists. The law is sound in principle and will justify itself in practice, and on account of the growing menace and disastrous effects of the narcotic drug habit it is necessary that the medical profession shall forego some slight modicum of its accustomed freedom from regulation, by checking up the prescription and use of the drug. This is required by the British Government in India and the other British possessions in the far East.

No prescription for any of the prohibited drugs, or in which they form a part, shall be written until after a physical examination of the patient, and it must contain the name, address and telephone call of the physician, the name, age and address of the patient, and the date of issue.

The drug shall not be sold at retail or to the consumer except on prescription, and if the prescription calls for more than a certain amount named in the statute the pharmacist or druggist must verify the correctness of the amount called for "by telephone or otherwise." Such prescriptions can be filled only once and only within ten days after its date, and shall be filed by the pharmacist or druggist and given a consecutive number on his file with the name and address of the purchaser making the purchase and the date upon which the sale was made; and he shall place upon the package containing the medicine a label, or deliver a certificate, stating the name and address of the person selling the drug, the name and address of the physician prescribing it, and the name and address of the purchaser. The physician, druggist, pharmacist, veterinarian and dentist shall keep a written record of the name and address of each person to whom he administers or disposes, or sells or delivers in any way any of the prohibited drugs.

No hypodermic syringe or hypodermic needle shall be sold at retail except upon the order of a duly licensed physician or veterinarian, and the seller shall enter in a book kept for that purpose the date of the sale, the name and address of the purchaser, and a description of the instrument sold.

The manufacturer or wholesaler of the prohibited drug shall sell only to manufacturing pharmacists, or chemists, or wholesaler, or retail pharmacists or druggists, or to hospitals, colleges, scientific or public institutions, and then only upon a written order upon an official order blank, which shall be retained and filed by the wholesaler or manufacturer, and an entry made on his books giving the date of sale, the name and address of the purchaser, and the name of person making the sale.

Possession of any of the prohibited drugs by "any person other than a manufacturer of any of the drugs, a wholesale dealer, a licensed pharmacist, a licensed druggist, physician, veterinarian or dentist, except as authorized by this law, is a misdemeanor."

Provision is made for the trial, conviction and commitment of habitual narcotic drug users to a hospital or other institution licensed by the State.

Provision is made that if a physician, dentist, veterinarian, pharmacist, or registered nurse, is proven to be "addicted to the use of any narcotic habit-forming drug," that his license shall be revoked for one year, and until he shall fully recover. A violation of any of the provisions of this law is a misdemeanor punishable by one year in the penitentiary, or by a fine of $500, or by both, and in case a physician, dentist, veterinarian, pharmacist, or registered nurse is convicted of a violation of any of the provisions of this law his license to practice may be revoked after notice and a hearing.

